# Application of medical gases in the field of neurobiology

**DOI:** 10.1186/2045-9912-1-13

**Published:** 2011-06-27

**Authors:** Wenwu Liu, Nikan Khatibi, Aishwarya Sridharan, John H Zhang

**Affiliations:** 1 Department of Diving Medicine, The Second Military Medical University, Shanghai, China; 2 Department of Anesthesiology, Loma Linda Medical Center, Loma Linda, California, USA; 3 Department of Neurosurgery, Loma Linda Medical Center, Loma Linda, California, USA

**Keywords:** Medical gases, Gas pre-conditioning, oxygen therapy, hydrogen therapy

## Abstract

Medical gases are pharmaceutical molecules which offer solutions to a wide array of medical needs. This can range from use in burn and stroke victims to hypoxia therapy in children. More specifically however, gases such as oxygen, helium, xenon, and hydrogen have recently come under increased exploration for their potential theraputic use with various brain disease states including hypoxia-ischemia, cerebral hemorrhages, and traumatic brain injuries. As a result, this article will review the various advances in medical gas research and discuss the potential therapeutic applications and mechanisms with regards to the field of neurobiology.

## Introduction

Medical gases, pharmaceutical molecules which offer solutions to a wide array of medical needs, range from traditional gases (oxygen and nitrous oxide) to gases like nitric oxide, carbon monoxide, and hydrogen sulfide--all of which have been recently shown to behave as biological messenger molecules [1]. Some gases, such as helium and xenon, have even been shown to be neuroprotective following various brain injuries such as acute ischemic stroke, perinatal hypoxia-ischemia, traumatic brain injury, and cardiopulmonary bypass-induced neurologic and neurocognitive dysfunctions [2-4]. In this paper, we will briefly introduce and review the history of oxygen, helium, xenon, and hydrogen gas, and discuss the various therapeutic mechanisms that have been proposed in the current literature.

### Oxygen

Air is composed of 78% nitrogen, 21% oxygen, and less than 1% of other gases. If more oxygen is needed, hyperoxia can be induced to increase the fraction of inhaled oxygen, and therefore the diffusion of oxygen through blood. This can be achieved under normobaric, or hyperbaric conditions. Normobaric hyperoxia (normobaric oxygen, NBO) is applied via a wide variety of masks that allow delivery of inspired oxygen ranging from 24% to 90%. Higher concentrations can be delivered via masks with reservoirs, tightly fitting continuous positive airway pressure-type masks, or through mechanical ventilation. In contrast, under hyperbaric conditions, one can breathe 100% oxygen (small chamber for single occupant), or breathe compressed air and 100% oxygen intermittently through a mask or hood (large multiplace hyperbaric chamber).

#### Mechanism underlying the theraputic effects of hyperoxia

Hyperoxia is an attractive therapeutic option because it has several properties of an 'ideal' protective agent. Unlike most pharmaceutical drugs, oxygen is simple to administer, easily diffuses to target tissues, is well tolerated, can be delivered in 100% concentrations without significant side effects, and can theoretically be combined with other treatments.

**Figure 1:**
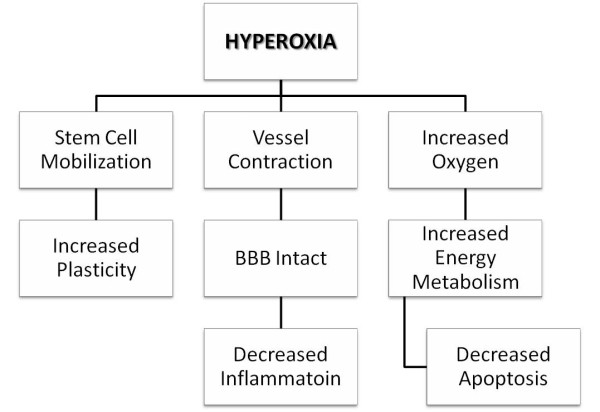
**Mechanisms underlying the protective effects of hyperoxia**. The mechanisms underlying the therapeutic effects of hyperoxia are quite complex, with a variety of mechanisms ranging from stem cell mobilization to enhancement of the neuroplasticity process under investigation.

To date, the mechanisms underlying the therapeutic effects of hyperoxia are quite complex, with a variety of mechanisms under investigation (Figure 1). According to some studies, hyperoxia has been shown to modulate aerobic metabolism, and to regulate blood flow via vasoreactivity. At the same time, the changes in aerobic metabolism can also regulate blood flow in the body. Therefore, although described independently, the mechanisms underlying the therapeutic potential of hyperoxia are vast and influence each other, to a certain degree.

#### Hyperoxia increases oxygen supply

Delivery of oxygen to tissues depends on adequate ventilation, gas exchange, circulatory distribution, and the partial pressure of inhaled oxygen. At normal sea levels, the partial pressure of oxygen (pO_2_) of inspired air is around 160 mmHg. This progressively drops as oxygen is carried further in the body. In other words, the first drop occurs in the lungs because of water vapor and diffusion, then in the vasculature leaving the alveolar capillaries, where the pO_2 _is around 104 mmHg, as it moves towards organs and tissues for perfusion. The diffusion distance of oxygen in a tissue is approximately 100~200 μm and an oxygen partial pressure of almost zero has been reported at about 100 μm from blood vessels [5,6].

It is also important to recall that the hemoglobin dissociation curve shows hemoglobin to be 100% saturated when the partial pressure of arterial oxygen (PaO_2_) is approximately 80 mmHg. Under conditions of complete saturation, as is the case with 100% oxygen administration under normobaric conditions, each gram of healthy hemoglobin contains 1.39 ml of oxygen, which accounts for only a small increase in the oxygen content of arterial blood.

Additionally, the amount of oxygen physically dissolved in the blood also increases proportionally with the partial pressure of ambient oxygen. The amount of oxygen dissolved in plasma is negligible under physiological oxygen tensions, but normobaric hyperoxia may increase the amount of dissolved oxygen by approximately 0.5~1% while hyperbaric oxygen can increase it considerably more. Inhalation of 100% oxygen at normal atmospheric pressure yields a 5-to 7-fold increase in arterial blood oxygen tension, which can only satisfy one-third of the oxygen requirements of resting tissue due to the low plasma solubility of oxygen. However, under hyperbaric conditions (such as 3 atmosphere absolute), the arterial blood oxygen tension may reach values close to 2,000 mm Hg, resulting in levels of plasma dissolved oxygen (about 6%) that can meet the average requirements of resting tissues, without a contribution from oxygen bound to hemoglobin [7].

The marked increase in oxygen tension gradient from the capillary blood to tissue cells is a key mechanism by which hyperoxygenation of arterial blood can improve effective cellular oxygenation, even at a low rate of tissue perfusion. The increased oxygen supply during normobaric hyperoxia or hyperbaric hyperoxia contributes to bactericidal effects within the tissue. Anaerobic microorganisms, in particular, do not have defense mechanisms against the increased production of reactive oxygen species (ROS) in hyperoxic environments [8,9].

#### Hyperoxia on the hemodynamics

In healthy animals and humans, oxygen inhalation can increase the total peripheral vascular resistance, secondary to systemic peripheral vasoconstriction. This results in a temporary increase in blood pressure, which is rapidly counterbalanced by a decrease in heart rate and cardiac output [10].

The brain has an extremely high metabolic rate; its oxygen demand exceeds that of all organs except the heart. Despite its relative small size (2% of total body weight), the brain receives approximately 20% of the cardiac output, and accounts for about 20% of the body's oxygen consumption. Over a normal physiological range of PaO_2 _(7 ± 13.33 kPa), there is little change in cerebral blood flow (CBF). Changes in oxygen tension are a potent stimulus for arterial dilatation/contraction. Rodent [11] and human [12] studies have shown that hyperoxia induces beneficial hemodynamic effects.

Kety and Schmidt originally described a reduction of 13% in CBF, and a moderate increase in the cerebrovascular resistance of young male volunteers inhaling 85-100% oxygen [13]. Omae and colleagues confirmed that increases in PaO_2 _led to a significant reduction in the flow velocity within the middle cerebral artery. However, when oxygen was increased from 100% at 1 ATA to 100% oxygen at 2 ATA, there were no further reductions in flow velocity within the middle cerebral artery [14].

Additionally, the increase in the mean partial pressure of oxygen in the tissues by hyperoxia has significant heterogeneities. The increase in heterogeneity is speculated to be a result of the redistribution of blood flow, with vasoconstriction in some areas and shunting in others. Under pathological conditions, hyperoxia may lead to the opening of collaterals, or a 'reverse steal' effect through diversion of blood from non-injured tissues into injured tissues. The unique combination of hyperoxia-induced vasoconstriction and high arterial blood oxygen decreases the vasogenic component of increased tissue hydrostatic pressure while preserving a high blood-to-tissue oxygen partial pressure gradient.

In the past, various mediators and mechanisms, such as increased effects of serotonin, nitric oxide synthase inhibition, inhibition of endothelial prostaglandin synthesis and increased leukotriene production, have been shown to play a key role in cerebral oxygen vasoreactivity [15].

#### Hyperoxia on energy metabolism

The downstream molecular effects of improved oxygenation are largely hypothetical. Increased aerobic metabolism leads to an increased production of ATP during aerobic cellular respiration in the mitochondria.

This hypothesis is indirectly supported by measurements of lactate and pyruvate in the cerebrospinal fluid (CSF). Daugherty et al investigated the oxygen consumption and mitochondrial redox potential during oxygen therapy [16]. Results showed that hyperbaric oxygen (HBO) not only induced an increase in oxygen consumption in both injured and sham-injured animals, but also enhanced the recovery of mitochondrial function [16]. Rockswold et al also revealed that HBO may improve aerobic metabolism by increasing the cerebral metabolic rate of oxygen and decreasing the lactate levels in CSF [17]. A more recent study revealed significantly greater improvements in the cerebral blood flow, cerebral metabolic rate of oxygen, microdialysate lactate, and the lactate/pyruvate (L/P) ratio when the partial pressure of oxygen in the brain tissue was ≥ 200 mm Hg, which is achieved during HBO or normobaric oxygen treatment [18]. Additionally, they found the effect of HBO on treatment to be more robust than that of normobaric oxygen on oxidative cerebral metabolism [18]. Normobaric hyperoxia, which increases brain tissue oxygenation, has a variable effect on the lactate and lactate/pyruvate ratio in addition to having a preferential metabolic benefit [19]. By using early high resolution positron emission tomography, Lou et al showed that exposure of the ischemic brain to HBO can partially reverse the downward trend for glucose utilization in the ischemic core, which might explain the reported benefits of early HBO therapy on permanent cerebral ischemia [20].

#### Hyperoxia on the inflammatory process

HBO contributes to curtail inflammation through various anti-inflammatory mechanisms. Following injury, white blood cells can migrate to the injured site producing inflammation. This is characterized by upregulation of adhesion molecules and chemokines, which play important roles in inflammatory migration. In this regard, HBO has been shown to decrease the rolling and adhesion mechanisms of polymorphonuclear leukocytes (PMNL), mediated by β2 integrin glycoproteins CD11/CD18, in the microcirculation following injuries [21]. Hyperoxia also reduces the expression of the endothelial adhesion molecules E-selectin [22] and intracellular adhesion molecule-1 (ICAM-1) [23]. Additionally, hyperoxia induces the production of endothelial NO synthase (eNOS) which synthesizes nitric oxide (NO), a protein which contributes to the reduction of PMNL adhesion to human vascular endothelial cell [24,25]. Additionally, hyperoxia reduces the mRNA and protein levels of cyclooxygenase-2 (COX-2), a key enzyme of prostanoid metabolism which also has a predominantly negative effect on inflammation induced by experimental ischemia [26]. By using directed microarray analysis, Rink et al showed that supplemental oxygen limited leukocyte accumulation at the infarct site by attenuation of the stroke-inducible pro-inflammatory chemokine response [27].

#### Hyperoxia on delayed cell death

Previous research suggests that hyperoxia can inhibit apoptosis. In several studies, HBO-treated rodents had reduced apoptotic markers in neonatal hypoxia-ischemia, as well as focal and global cerebral ischemia models [28,29]. Additionally, Henninger et al [30] reported that normobaric oxygen treatment reduces apoptotic cell death in the ischemic hemisphere.

#### Hyperoxia on vascular permeability

Hyperoxia has also been shown to preserve the integrity of the blood-brain barrier (BBB) by reducing vascular permeability. Previous studies have shown that early intra-ischemic HBO treatment reduces the BBB disruption, hemorrhagic transformation, and subsequent mortality following focal cerebral ischemia [31,32]. Additionally, Sun et al used MRI to demonstrate that hyperbaric and normboaric oxygen treatments significantly reduce post-ischemic BBB permeability on post-contrast T1-weighted images in middle cerebral artery occlusion (MCAO) models [33].

Protection of the BBB by hyperoxia may be related to the reduction of matrix metalloproteinases (MMPs), which are predominant enzymes involved in the degradation of the components of the vascular basement membrane [34]. This effect may be mediated by NADPH oxidase [35] and/or hypoxia-inducible factor (HIF) [36] because reduction of NADPH oxidase after hyperoxia results in the decreased production of ROS, which play an important role in the activation of MMPs [37]. Moreover, HIF-1 has been implicated in the control of MMPs, including MMP-2 (gelatinase A) and MMP-9 (gelatinase B), which can degrade components of the extracellular matrix surrounding cerebral blood vessels [38].

#### Hyperoxia on the plasticity process

Following an initial cerebral injury, post-acute brain plasticity is critical for determination of functional brain improvement. The absence of neuroanatomical plasticity following cerebral injury is attributable to several factors, including glial scars, lack of neurotrophins, and growth inhibitory molecules. Neurite outgrowth inhibitor (Nogo-A), one of the most powerful growth inhibitors, is related to the plasticity of the central nervous system. Zhou et al revealed that HBO significantly decreased the levels of Nogo-A, and Ras homolog gene family, member A (RhoA) during an ischemic injury in the cortex, which might partially contribute to the improvement of neurological function [39]. Garcia et al also showed that a transient acute hyperoxia stimulus, whether caused by breathing HBO or reoxygenation following hypoxia, is a powerful stimulant for orthodromic activity and neural plasticity in the CA1 hippocampus [40]. Additionally, hyperbaric oxygen treatment promotes neural plasticity by increasing Map-2 expression and decreaseing GFAP [41].

#### Neuroclinical application of oxygen therapy

Oxygen therapy remains a cornerstone of modern medical practice and many of its physiological actions have already been elucidated. With regards to the field of neurology, oxygen therapy has been studied in stroke and traumatic brain injury (TBI) cases due to the prevalence of both diseases.

#### Normobaric hyperoxia on traumatic brain injury

In 1998, Thiagarajan et al applied hyperoxia in TBI patients undergoing hyperventilation. Their results showed that increases in the PaO_2 _above normal levels could offset the deleterious effects of hyperventilation on jugular bulb oxygen saturation and arteriovenous oxygenation content difference in patients with head injuries [42]. To this date, the most cited study was conducted by Tolias et al [43]. This was a prospective study where 52 patients with severe TBI were treated with a FiO_2 _(fraction of inspired oxygen) of 100% for 24 h within 6 h of admission. When compared with 112 patients in a historical cohort study, patients treated with 100% FiO_2 _had increased glucose levels and decreased glutamate and lactate levels. Microdialysis also showed reductions in the lactate/glucose and lactate/pyruvate ratios. Additionally, intracranial pressure was reduced significantly in the treatment group, while there were no changes in cerebral perfusion pressure. Unfortunately, one of the limitations of this study was the potential for hyperoxia-induced pulmonary toxicity due to the longevity of the treatment. Magnoni et al performed a variation of the Tolias study by administering 100% FiO2 to patients with TBI for 3 h [44]. Interestingly, similar results were found except that the lactate/pyruvate ratio, and the levels of glucose and glutamate remained unchanged.

In a study by Nortje et al, oxygen-15 positron emission tomography (15O-PET) analysis revealed that normobaric hyperoxia administered following TBI conferred a preferential metabolic advantage to the "at risk" peri-lesional tissue, as measured by improved local cerebral metabolic rate of O_2 _(CMRO_2_) and brain tissue oxygen tension (PbtO_2_) [19]. However, a study from Diringer et al showed that 1 h hyperoxic treatment was not sufficient to exert positive effects on cerebral O_2 _metabolism [45]. Cumulative analysis of these studies delineates the beneficial effects of normobaric hyperoxia on TBI. In fact, normobaric hyperoxia may offer a simple, minimally invasive, and easily applicable adjunct in the early management of TBI.

#### Normobaric hyperoxia on stroke

Several groups have become interested in NBO because it offers distinct advantages over HBO: it is widely available, simple to administer, inexpensive and most importantly, can be started very quickly by paramedics after stroke onset.

Recent rodent studies using the transient middle cerebral artery ischemia-reperfusion model have shown that NBO improves pathological, neurobehavioural, and neuroimaging outcomes following stroke. Singhal et al showed that NBO therapy administered during ischemia and during the immediate post-reperfusion period results in a 70% reduction of hemispheric infarct volumes in rats [11,46]. The therapeutic time window for NBO in rodents is short (approximately 30~45 min); Singhal et al used serial diffusion-weighted imaging (DWI) to show that initiating treatment at earlier time points enhances the degree of neuroprotection and attenuates the severity and the volume of ischemic injury.

The first study on normobaric hyperoxia in stroke was an observational study led by Ronning and Guldvog. Their results showed that the one-year survival was higher in room-air treated control patients with mild-moderate stroke when compared to those treated with 3 L/min nasal oxygen [47]. However, there were a lot of shortcomings in this study. For example, the time to treatment was relatively late as 18% patients did not receive the assigned 'therapy', 12.7% had hemorrhagic stroke.

The first therapeutic trial of NBO (45 L/min oxygen via a facemask) was carried out by Singhal et al [12]. Results showed that NBO-treated stroke patients had improved National Institutes of Health Stroke Scale (NIHSS) scores, reduced growth of DWI lesion volumes, and an increase in the volume of 'penumbral' tissue. NBO therapy also resulted in a marked improvement in the visibilty of DWI lesions. Additionally, the percentage of MRI voxels improving from baseline "ischemic" to 4-h "non-ischemic" values tended to be higher in NBO-treated patients. Cerebral blood volume and blood flow within ischemic regions also improved with hyperoxia. These findings show that initation of NBO treatment within 12 h of stroke transiently improves clinical features as well as MRI parameters of ischemia.

Building on their previous work, Singhal et al. performed multivoxel magnetic resonance spectroscopic imaging and diffusion/perfusion MRI in stroke patients exposed to normobaric hyperoxia (45 L/min oxygen through a face mask for 8 h), or room air. Imaging, performed before, during, and after therapy, helped conclude that normobaric hyperoxia improves aerobic metabolism [48]. Additionally, a similar study showed that administration of 40% FIO_2 _(via venture masks) to patients with middle cerebral artery strokes decreased stroke-related complications as well as mortality [49]. However, these studies need to be further validated due to their small sample size.

#### Hyperbaric oxygen on traumatic brain injury

Hyperbaric oxygen treatments typically involve pressurizations between 1.5 and 3.0 ATA for 60 to 120 min daily. A number of studies have demonstrated the effectiveness of HBO in the treatment of TBI using the Glasgow Coma Scale (GCS) and Glasgow Outcome Scale (GOS) to evaluate outcomes. These studies have demonstrated that hyperbaric oxygen treatment increases GCS and GOS scores [50-53]. Additionally, Rockswold et al showed that HBO therapy could also increase cerebral metabolic rate of oxygen (CMRO_2_) and decrease lactate levels in the CSF, thereby improving aerobic metabolism in patients with severe brain injuries [16,17].

Shi et al used single photon emissions computed tomography (SPECT) images and computed tomography scans (CT) to evaluate the use of HBO therapy on neuropsychiatric disorders following TBI. The results, which revealed that hyperbaric oxygen treatment improved regional cerebral blood flow, were consistent with another study conducted by by Sukoff et al in 1982 [54].

With regards to CSF pressures, Mogami et al. found that CSF pressures of severely brain-injured patients decreased during the beginning of treatment, stayed at a low level during treatment, and rebounded post-treatment [55]. These findings are similar to what Hayakawa et al found a few years later [56].

Although much there have been studies on the efficacy of HBO therapy following TBI, there is insufficient evidence to prove or disprove HBO's therapeutic effectiveness. The evidence indicates that there is a small chance of a mortality benefit with HBO therapy in TBI patients, depending on the subgroup selection. HBO's effect on functional status as well as the incidence and clinical significance of its adverse effects are unclear. In four reviews, the authors consistently concluded that the routine adjunctive use of HBO therapy in patients with TBI cannot be justified due to various limitations in the existing studies, such as a small number of studies, modest numbers of patients, and the methodological and reporting inadequacies of the primary studies [57-60].

#### Hyperbaric oxygen on ischemic stroke

In 1960, Boerema et al showed, for the very first time, that exsanguinated pigs could survive by placing them in a HBO chamber [61]. This study intensified efforts to use HBO to treat organ ischemia. Unfortunately, 3 years later, Jacobson and Lawson reported no benefit following HBO at 2.0 ATA following MCAO in dogs [62]. But since the 1960's, numerous animal studies and clinical trials have documented substantial benefits with HBO in animals and/or patients with transient and permanent focal stroke.

Typical HBO therapy regimens use 100% oxygen at 1.5~2.5 atm pressures for 30~90 min with the time between sessions and the number of sessions varying widely. Recent animal data indicates that oxygen therapy is most effective if applied early, for short durations, and that long-term benefit can be maximized if the tissue is reperfused [63-66]. Delayed and repetitive HBO treatments can expand the therapeutic window in rats with focal cerebral ischemia [67] and can promote neurological recovery in the acute setting, while reducing the frequency of recurrent strokes [68-71].

To this date, three randomized clinical trials using HBO therapy in stroke have been published [72-74], and all three trials have reported negative results. The first trial by Anderson et al was interrupted early due to concerns regarding randomization. Interestingly enough, prior to its discontinuation, the interim analysis revealed a trend towards improvement in neurological examination and infarct volumes in the HBO therapy patient group. In the second trial, conducted by Nighoghossian et al, the Rankin score, Trouillas score, and the Orgogozo scale score, were used to evaluate the effectivess of HBO therapy. The results showed that Orgogozo and Trouillas scores at 1 year were significantly better in the HBO group; however, a comparison of the pre-and post-therapy differences between the HBO groups at 6 months and 1 year did not show statistical significance on any of the scoring systems. In the final clinical study, conducted by Rusyniak et al, the results after 3 months revealed that the neurological outcome scores (NIHSS, Rankin scale, Barthel index and Glasgow outcome scale) were significantly better in the sham group (100% oxygen) compared to the HBO-treated group in all scales except the Barthel index. These clinical studies suggested that HBO therapy does not benefit to ischemic patients and may even be harmful in stroke.

Following these three clinical trials, Bennett et al conducted a meta-analysis and review of the trials, and suggested that existing data do not justify the use of HBO therapy in stroke patients, and that the lack of a significant effect may be related to the small number of patients, delays in initiating HBO therapy, and absence of successful blinding [75].

### Hyperoxia Pre-Conditioning

Pre-conditioning is a process by which an organism's exposure to a stress/stimulus allows it be more resilient against the same stimulus during future encounters. One of the first studies, led by Dahl et al, found that rats pre-conditioned with a brief episode of anoxia could survive longer (90 sec) than control animals (60 sec) following a prolonged exposure to anoxia [76]. In this study, they speculated that energy metabolism played an important role in the prolonged survival time of the preconditioned rats. This effect was later confirmed in adult brain slices with pre-exposure to a short (5 min) anoxic episode [77]. That same year, Geft et al introduced brief periodic coronary occlusions up to 18 times by inflating and deflating the balloon for periods of 15, 10, or 5 min, followed by 15-min periods of reperfusion. Their findings suggested that intermittent reperfusion had a beneficial effect and may prevent necrosis, even when total occlusion time exceeds 200 min [78]. It wasn't until 1986 that Murry et al proposed the concept of pre-conditioning, and confirmed the protective effects of ischemia pre-conditioning on myocardial ischemia [79]. A turning point came when Kitagawa et al documented the ability of brief bilateral carotid occlusion to protect gerbil hippocampal CA1 pyramidal neurons against prolonged global ischemia; these authors coined the term 'ischemic tolerance' to describe the phenomenon [80]. To this date, the protective effects of ischemic pre-conditioning have been confirmed following ischemic exposures to the heart [81], brain [82], lung [83], kidney [84], and liver [85].

Compared with hypoxic pre-conditioning, hyperoxia pre-conditioning via HBO or NBO pre-conditioning seems to be a safer and more readily available in clinical practice. In 1986, Martin et al found pre-treatment with endotoxin and pre-conditioning with hyperoxia could reduce the inflammatory response in paraquat-induced neutrophil alveolitis [86]. Four years later, D'Brot et al suggested that prior exposure to alveolar hyperoxia could prevent the hypoxia-induced enhancement of bronchial reactivity to carbachol and histamine, which involves a cyclooxygenase-dependent mechanism [87]. In 1996, a Japanese group showed that repeated exposures to hyperbaric oxygen (100% oxygen at 2ATA for 1 h each) could increase the tolerance of the brain against ischemic neuronal damage, in which the induction of HSP-72 synthesis plays an important role [88]. Since then, the protective effects of hyperoxia pre-conditioning, especially the hyperbaric oxygen pre-conditioning, have been investigated in a lot of animal models.

#### Mechanisms underlying the protective effects of hyperoxia pre-conditioning

**Figure 2:**
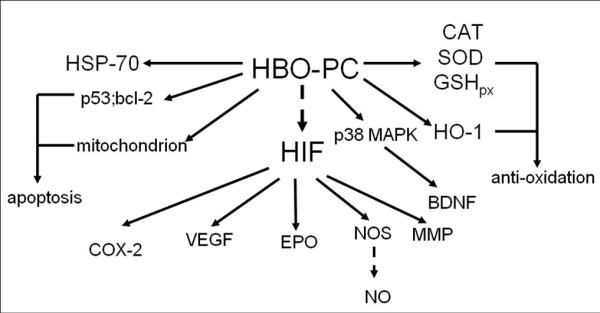
**Mechanisms Underlying Hyperoxia Pre-conditioning**. The protective effects of hyperoxia pre-conditioning can be attributed to a variety of mechanisms ranging from VEGF activaition to anti-oxidant production.

Previous studies have suggested that the protective effects of hyperbaric oxygen pre-conditioning could be attributed heat shock proteins (HSPs) [88,89]. However, Vince et al recently suggested that the benefits of HBO pre-conditioning may not be due to inducement of HSP expression in circulating blood cells, but might involve an enhancement of the stress response [90] (Figure 2).

Exposure to hyperbaric oxygen can generate oxidative stress [91], which can subsequently activate antioxidants that could confer protective effects. Although excessive oxidative stress can damage tissues, previous works have shown that pre-conditioning with mild hyperoxia can produce an increased antioxidant response [92-98].

Additionally, intermittent exposures to hyperoxia may produce a temporary 'hypoxic environment' between two hyperoxia exposures which may induce the expression and/or activity of a key oxygen sensor known as hypoxia-inducible factor-1 (HIF1) [99,100]. The increased HIF1 level may regulate the expressions of its downstream genes including vascular endothelial growth factor (VEGF) [101], COX-2 [102,103], erythropoietin (EPO) [100], nitric oxide synthase (NOS) [104] and MMP [105]. Moreover, hyperoxia pre-conditioning can suppress the activity of p38 mitogen activated protein kinase (MAPK) [106-108] and induce expression of brain-derived neurotrophic factors [106], which can also induce the expression of HIF-1 [109].

Other important mechanisms of protection from hyperoxia pre-conditioning include a reduction in apoptosis, especially following ischemic brain injury [106,110-112]. Within the past decade, researchers have also investigated the role of transporters and key inflammatory cytokines, such as glutamate transporters, tumor necrosis factor-α, nuclear factor-κB, ribosomal protein S6 kinases, and mitochondrial ATP-sensitive potassium, in the protective effects of hyperoxia pre-conditioning [89,113-116]. Some have even shown that the genes/proteins relevant to neurotrophin and the inflammatory-immune system may be involved in HBO-induced protection [117].

#### Application of hyperoxia pre-conditioning

The neuroprotective effects of hyperoxia have been investigated in various models, including global ischemia [105], forebrain ischemia [107], surgical brain injury [102], ischemia-reperfusion injury [97,110], neonatal hypoxia-ischemia [111], intracerebral hemorrhage [89], and spinal cord ischemia [118].

#### Oxygen toxicity

Oxygen toxicity is a legitimate concern with regards to the clinical application of hyperbaric oxygen. Once oxygen is applied at a relatively high pressure for prolonged periods of time, various systems and organs can be negatively affected including the central nervous and pulmonary systems [119]. In clinical practice, oxygen is usually applied at 2~3 ATA and the duration of oxygen treatment ranges from 60 min to 2 h. Moreover, the oxygen inhalation is frequently interrupted by inhalation of air at corresponding pressures. Under these conditions, oxygen toxicity is rare. However, in certain environments, oxygen may be applied at high pressures and for longer durations such as in the treatment of central nervous system decompression sickness/dysbarism [120] and with preemies [121]. Under this condition, the oxygen partial pressure and treatment duration should be closely monitored to minimize the potential for the oxygen toxicity. Although some attempts to minimize the detrimental effects of oxygen exposure with anti-oxidants have been made, nothing has been confirmed [122].

### Helium

#### Introduction

In addition to being a well-defined noble gas, helium is the second most abundant element in the universe, and accounts for 24% of the elemental mass of our galaxy. Helium has many applications in cryogenics, the cooling of superconducting magnets, MRI scanners, arc welding, and can also be used as a gas pressureizer. Helium has traditionally been used to help decrease the incidence of decompression sickness during deep sea diving [123,124].

In 1934, Barach proposed the use of helium as a new therapeutic gas [125], and subsequently proposed heliox (helium/oxygen gas mixture) as a potential therapy for asthma exacerbations [126]. Since then, studies have increasingly demonstrated the benefits of heliox inhalational therapy. Within the past decade, heliox has become a focus of renewed interest as treatment for acute asthma exacerbations [127], bronchiolitis [128], chronic obstructive pulmonary disease (COPD) [129]. Heliox has also been recognized as an important adjunct in the treatment of severe asthma exacerbations in the 2007 guidelines published by the National Heart, Lung, and Blood Institute. During treatment of severe asthma exacerbations in children, inhalational heliox therapy led to significant improvements in pulsus paradoxus, peak flow, and dyspnea [130]. The therapeutic effects of helium may be attributed to an increased flow rate and decreased turbulent flow, resulting in deeper penetration of gases to the distal alveoli, higher minute volumes, and improved ventilation. Additionally, helium has been shown to have a high diffusion coefficient for carbon dioxide relative to oxygen. Correspondingly, this diffusion coefficient may facilitate an environment for increased pulmonary exhalation of trapped carbon dioxide (carbon dioxide washout effect), which is another important factor underlying the therapeutic effect of heliox [131].

Despite the first clinical success of heliox use in 1935 and an ample published support over the last decade, heliox is still not widely available in acute care facilities. According to many high level researchers and clinicians, much of the evidence for its use is anecdotal with a paucity of level 1 data. Further studies with homogeneous logistics, a larger patient population, and a more definitive outcome measure are needed to clearly define the utility of heliox.

#### Biological effects of helium and potential mechanisms

Helium has been reported to increase the coronary collateral circulation [132] and enhance the vasodilatory effects of inhaled nitric oxide on pulmonary vessels [133] (Table 1). In healthy men, heliox improved exercise tolerance while reducing rising lactate levels, without a significant change in vitals i.e. blood pressure or heart rate [134]. In 2007, Pagel et al applied three cycles of 70% He-30% O_2 _for 5 min interspersed with 5 min of 70% N_2_-30% O_2 _before opening up a left anterior descending coronary artery occlusion. Results showed that pre-conditioning with helium could provide cardioprotection by activating pro-survival signaling kinases and inhibiting the opening of the mitochondrial permeability transition pore (mPTP) [135]. In the following year, Heinen et al also applied heliox as a pre-conditioning modality in a rat heart ischemia/reperfusion model. Their results revealed that the cardioprotective effects of heliox pre-conditioning in young rats, which were mediated by mitochondrial uncoupling and Ca^2+^-sensitive potassium channel activation [136]. In a series of studies led by Pagel et al, researchers found that reactive oxygen species (including nitric oxide) [137,138], mitochondrial adenosine triphosphate-regulated potassium channels, mitochondrial permeability transition [139], and opioid receptors [140] played critical roles in the cardioprotective effects induced by helium pre-conditioning.

**Table 1:** Biological Effects and Potential Mechanisms of Helium

BIOLOGICAL EFFECTS	POTENTIAL MECHANISMS
**Coronary Collateral Circulation**	**Prosurvival Signaling Kinases **[137]
**Improve Exercise Tolerance**	**MPTP Opening **[139]
**Anti-Arrhythmic Effect**	**Mitochondrial Uncoupling **[136]
**Anti-Tumor Effect**	**Ca^2+^-Sensitive Potassium Channel **[136])
**Anti-Inflammation**	**Reactive Oxygen Species **[137,138]
**Hypothermia**	**Mitochondrial ATP-Regulated K^+ ^Channel **[139]
**High Pressure Nervous Syndrome**	**Mitochondrial Permeability Transition **[139]
**Neuroprotective Effect**	**Opioid Receptors **[140]
**Myocardioprotective Effect**	**COX-2 Activity **[23]

With regards to human clinical trials, Lucchinetti et al reported no protection with helium use. Specifically, 8 healthy adults were recruited to inhale helium at an end-tidal concentration of 50% from 15 min before ischemia to 5 min after the onset of reperfusion. Although their results indicated that inhaled helium diminishes the post-ischemic inflammatory reaction, human endothelium was not amenable to protection by helium at 50% *in vivo *[141].

#### Application of helium in neurology

Few studies have truly investigated the effects of heliox treatment or pre-conditioning in the nervous system. In 2007, Pan et al compared the efficacy of hyperoxia (100% oxygen) and that of heliox (30% oxygen/70% helium) during the ischemia/reperfusion process in the brain [142]. Their results showed that heliox was superior to hyperoxia with regards to infarct volume reductions and improvements in neurological deficits at 24 h. One year later, Coburn et al reported that helium could exert neuroprotective effects at elevated pressures in an *in vitro *model of traumatic brain injury [2]. However, the exact mechanism underlying the protective effects of heliox has not been reported.

These results seems to conflict with those in the study led by Pan et al, where body temperature, mean arterial blood pressure, heart rate, oxygen saturation and laser Doppler regional cerebral blood flow parameters were not significantly different before, during or after MCAO injury-neither between the control, hyperoxia, nor heliox groups. In addition, to this date two *in vitro *studies have refuted the protective effects of helium. They reported that heliox was detrimental to neurons [143], and human tubular kidney cells [144].

### Xenon

For decades, volatile anesthetics have been extensively studied with regards to their potential neuroprotective properties. A colorless, heavy, odorless noble gas, xenon has been of particular interest to researchers because of its possible neuroprotective properties.

#### Biological effects of xenon and potential mechanisms

Current evidence suggests that xenon induces anesthesia and exerts its analgesic actions by inhibiting the N-methyl-D-aspartate (NMDA) receptor signaling pathway [145,146]. Under conditions that mimic synapses of mammalian systems, non-NMDA receptors were found to be insensitive to xenon [147]. Interestingly, xenon inhibits the current generated when an artificial agonist (kainite) was directly applied to recombinant AMPA receptors; however, the sensitivity of these receptors to xenon was negligible when they were activated by the natural agonist (glutamate), to mimic synaptic conditions [147].

In addition, xenon can inhibit (α_4_)_2_(β_2_)_3 _nACh receptors expressed in *Xenopus oocytes*, whereas the α_4_β_4 _nACh receptor was only slightly affected [148]. Xenon also competitively blocks the 5HT3A receptor, which has been implicated in regulation of emesis and peripheral nociception, at clinically relevant concentrations [149,150]. Additionally, Xenon also activates TREK channels [151], which has a neuroprotective role during spinal chord ischemia (Heurteaux et al., 2004).

In addition to acting on receptors, xenon affects neurotransmitter release. In rats, xenon stimulates noradrenergic neurons in the hypothalamus more than nitrous oxide. *In vivo *microdialysis studies in the rat cerebral cortex revealed that xenon induced an initial increase in ACh release followed by a gradual decrease [152], while there were no xenon-induced effects on acetylcholinesterases *in vitro *[153]. Additional studies have demonstrated that xenon may act on the secondary messenger signaling pathway via increases in cyclic guanosine monophosphate (cGMP). Galley et al showed that xenon can increase cGMP levels in the spinal cord, brainstem, and hippocampus, without altering the activity of the neuronal nitric oxide synthase [154]. Furthermore, Petzelt et al showed that xenon can influence mechanisms regulating the Ca^2+ ^release channel on plasma membranes [155]. In rat brain synaptic plasma membranes, xenon inhibits plasma membrane calcium ATPase pump activity, resulting in an increase in neuronal Ca^2+ ^concentration and an altered excitability in these cells [156]. The decrease in regional CBF after xenon treatment [157] may help reduce intracranial pressure, and the regional cerebral metabolic rate for glucose [158].

In terms of the cardioprotective effects of xenon, studies have shown that it can stimulate several important intracellular prosurvival signaling kinases, including protein kinase C (PKC) [159,160], MAPK [159,161], HSP 27 [161], extracellular signal-regulated kinases 1/2 [162], Akt, GSK-3β, and Ca^2+^-induced mPTP [163]. Xenon's role in anti-apoptotic mechanisms also demonstrates its neuroprotective qualities [164,165].

Furthermore, studies have suggested that xenon plays an important role in the anti-inflammatory process. Xenon has been specifically shown to increase the removal of P-selectin glycoprotein ligand-1 (PSGL-1) and L-selectin from the neutrophil surface *in vitro*. Since both selectins are involved in the initial contact between neutrophils and endothelial cells, xenon may affect neutrophil adhesion to endothelium during ischemia/reperfusion injury [166]. Moreover, xenon has been shown to prevent TNF-α-induced expression of ICAM-1 and to decrease TNF-α-induced transcriptional activity of nuclear factor κB (NF-αB). However, xenon's protective effect is abolished by functional blockade of COX-2 during xenon-induced late myocardial pre-conditioning [167].

#### Application of xenon in neurology

Since the discovery of xenon as an NMDA receptor antagonist, there has been growing interest in its potential use as a neuroprotectant. Some of the features of xenon that specifically interest scientists and researchers include its rapid introduction into the brain, favorable hemodynamic profile with little or no toxicity, and its inability to be be metabolized [168] (Table 2).

**Table 2:** A Summary of the Neuroprotective Effects of Xenon

Model	Intervention	Results	Reference
NMDA, glutamate, or oxygen deprivation induced neuronal injury	Xenon saturated medium for 24 h (*in vitro*) 20%, 40%, 60%, 75% xenon (*in vivo*)	Xenon (60% atm) reduces LDH release to baseline with oxygen deprivation; xenon (75% atm) reduces LDH release by 80% with either NMDA-or glutamate-induced injury. *In vivo*, xenon exerts a concentration-dependent protective effect and reduces injury by 45% at the highest xenon concentration tested (75% atm).	[170]
Hypoxia damaged cortical neurons from rat embryos	Xenon saturated medium for 2 h	Complete protection against cellular damage and prevention of hypoxia-induced glutamate release	[171]
Hypoxia damaged PC-12 cells	Xenon saturated medium for up to120 min	Xenon results in complete protection against cellular damage and prevention of hypoxia-induced dopamine release in which intracellular Ca^2+^-ions evolve.	[172]
MCAO in mice	70%, 35% xenon during occlusion for 60 min	Xenon administration improves both functional and histological outcome	[173]
Neonatal HI	70%, 50% xenon immediately after insult for 3 h	Xenon administration commenced after hypoxia-ischemia in neonatal rats provides short-term neuroprotection	[174]
brain slices from rats (OGD) MCAO	15-75% xenon bubbled medium 50% xenon 2~3 h after MCAO	Xenon, administered at subanesthetic doses, offers global neuroprotection from reduction of neurotransmitter release induced by ischemia, reduces subsequent cell injury and neuronal death	[175]
NMA induced neuronal damage	70% xenon for 10 min at 3 h, 1, 2, 5, or 7 days before insult	Xenon alone does not induce changes, but reduces about 50% NMDA-induced cell loss as well as degenerating neurons, with the maximal neuroprotection at 7 days.	[176]
anesthetic-induced neuronal apoptosis in vivo and in vitro	75%, 60%, 30% xenon for 6 h	Xenon attenuates isoflurane-induced apoptosis.	[164]
nitrous oxide and isoflurane induced damage	70% xenon for 2 h	Xenon pre-treatment prevents nitrous oxide-and isoflurane-induced neuroapoptosis (*in vivo and in vitro*) and cognitive deterioration (*in vivo*)	[165]
OGD induced damage to neurons from neonatal mice	75% xenon + Dex (0.001~10 μM) for 6 h	Combination of Xenon and Dex offers neuroprotection additively *in vitro *and synergistically *in vivo*	[178]
neonatal HI	20-70% xenon for 90 min during hypoxia or 2, 24 h after hypoxia + hypothermia (30-37°C)	Xenon and hypothermia administered 4 h after hypoxic-ischemic injury in neonatal rats provides synergistic neuroprotection	[177]
OGD induced damage to neurons; neonatal HI	25~75% xenon for 120 min (*in vitro*); 70% xenon for 120 min (*in vivo*)	Prosurvival proteins Bcl-2 and brain-derived neurotrophic factor are upregulated by xenon treatment	[179]
OGD induced damage to neurons; neonatal HI	12.5~75% xenon for 2 h (*in vitro*); 20%, 75% for 2 h (*in vivo*)	Pre-conditioning with xenon and the combination of xenon and sevoflurane results in long-term functional neuroprotection associated with enhanced phosphorylated cyclic adenosine monophosphate response element binding protein signaling	[180]
MCAO in mice	70% xenon for 2 h	Xenon pre-conditioning improves histological and neurological functional outcome in both genders in a stroke model of mice in which HIF-1α and phosphoAkt evolve	[207]
OGD induced damage to neurons	75% xenon for 2 h	Xenon pre-conditioning clearly involves the activation of K_ATP _channels.	[208]

Xenon has been shown to be a remarkably potent neuroprotectant in a variety of models [169]. Its dose-dependent neuroprotective effects have been studied in rat neuronal-glial co-cultured with NMDA-induced toxicity, glutamate-induced toxicity, hypoxic injury, and oxygen-glucose deprivation [170-172]. The protective effects of xenon have also been identified in rodents subjected to focal ischemia and neonatal hypoxic-ischemic injury [173,174]. Additionally, at subanesthetic doses, xenon has been shown to offer global cerebral neuroprotection by reducing the ischemia-induced neurotransmitter release as well as subsequent cell injury and neuronal death that follows an ischemic attack. Maximal neuroprotection was obtained at a concentration dose of 50%, even when xenon was administered up to 4 h after intrastriatal NMDA injection and up to at least 2 h following the induction of transient brain ischemia [175].

In one study, Natale et al investigated the neuroprotective properties of xenon by evaluating its morphological changes on rat arcuate nucleus. After 3 h, 1, 2, 5, or 7 days from gas exposure, the neuronal damage was significantly reduced in the xenon treatment group. They concluded that xenon could be regarded as a promising neuroprotective agent [176]. In a neonatal hypoxia-ischemia model, Dingley et al showed that 3 h of xenon administration, following hypoxia-ischemia, provided short-term neuroprotection in neonatal rats [174].

Researchers have also explored the possibility of using xenon in combination with other therapeutic strategies to evaluate its possible synergistic neuroprotective capabilities. In one study, a combination of xenon and hypothermia, administered 4 h following hypoxic-ischemic injury, provided synergistic neuroprotection in neonatal rats [177]. Studies also revealed that the combination of xenon and dexamethasone could offer additive neuroprotection both *in vitro *and *in vivo *[178] (Table 2)

Recently, xenon has been investigated as a brain pre-conditioning agent. Ma et al observed that xenon-induced pre-treatment in neonatal hypoxic-ischemic rats decreased infarction size and improved neurological function [179]. Additional pre-conditioning studies with xenon alone and with the addition of sevoflurane resulted in long-term functional neuroprotection associated with enhanced phosphorylated cyclic adenosine monophosphate response element binding protein signaling [180]. Xenon pre-treatment prevented nitrous oxide-and isoflurane-induced neuroapoptosis (*in vivo *and *in vitro*) and cognitive deterioration (*in vivo*) [165].

### Hydrogen

The first element in the periodic table is hydrogen (H_2_). As the lightest and most abundant chemical element, hydrogen constitutes roughly 75% of the Universe's chemical elemental mass. In comparison, free hydrogen is rare on Earth, constituting less than 1 part per million in the atmosphere, with the majority found in water and organic compounds.

Hydrogen is continuously synthesized in human cells during fermentation of non-digestible carbohydrates by intestinal bacteriain the large intestine, and is later excreted as flatus [181,182]. This is the basis for the routinely-used breath hydrogen as a test for gastrointestinal transit. An estimated 14% to 20% of the total colonic H_2 _production was reported to be carried through the human bloodstream and subsequently released into the lungs [183]. Therefore, exhalation of hydrogen forms the basis for assessing gastrointestinal motility and transit time as well as the overgrowth of of small intestinal bacterial [184-186].

In 1975, Dole et al first applied hyperbaric hydrogen when treating mice with squamous cell carcinoma [187]. Since their results revealed significant regression of tumors, they speculated that hyperbaric hydrogen therapy may act as a free radical decay catalyzer and prove efficacious in other cancer types [187]. On the other hand, Kayar et al showed hydrogen gas is not oxidized by mammalian tissues under hyperbaric conditions and instead attributed its effect to other unknown properties [188]. It wasn't until 2001 that Gharib et al investigated the anti-inflammatory properties of hydrogen in a schistosomiasis-associated chronic liver inflammation model. The results revealed that hyperbaric hydrogen at 0.7 MPa for 2 weeks had significant protective effects on liver injury by decreasing fibrosis while improving hemodynamics and the activity of antioxidant enzymes [189]. However, few studies emphasized the therapeutic effects of hydrogen before 2007. This was when a Japanese group conducted a series of experiments investigating the biological effects and therapeutic potency of hydrogen (~4%) [190]. Since then, a large number of studies have explored the therapeutic effects of hydrogen in different tissues including the brain, heart, kidney, lung, and liver.

#### Mechanisms underlying the bioeffects of hydrogen

One of hydrogen's antioxidant capabilities is as a free radical scavenger. It selectively reduces the levels of hydroxyl radicals (·OH), which are mainly generated through the Fenton reaction and peroxynitrite (ONOO^-^) *in vitro *[190], and are both strong oxidants that react indiscriminately with nucleic acids, lipids, and proteins to cause DNA fragmentation, lipid peroxidation, and protein inactivation. Another possible mechanism underlying the cellular protection afforded by hydrogen may be its ability to increase antioxidant enzymes such as catalase, superoxide dismutase, and heme oxygenase-1 [191,192]. Some studies have suggested that hydrogen may have antiapoptotic properties, and can inhibit caspace-3 activation [193].

Therefore, hydrogen has several potential advantages over current pharmacological due to its safety and therapeutic potency. Hydrogen's ability to easily traverse the brain-blood barrier allows it to be safely administered to human patients. Furthermore, it is capable of rapidly diffusing through membrane compartments to gain ready access to the cytosol, mitochondria, and nucleus. Finally, hydrogen reduces detrimental hydroxyl radicals and peroxynitrite, but does not compromise essential homeostatic mechanisms dependent on ROS.

#### Application of hydrogen in neurology

The potential benefits of hydrogen gas on various brain pathologies have been investigated since 2007, when the first hydrogen paper was published (Table 3). In China [194,195] researchers found that inhalation of 2% hydrogen and injection of hydrogen-saturated saline following hypoxic-ischemic brain injuries evoked neuroprotection via inhibition of neuronal apoptosis and reduction of caspase-3 and caspase-12 activities. These short-term effects also translated into long-term improvements in when neurobehavioral functions were tested 5 weeks following the injury [195]. However, Matchett et al reported conflicting results [196] in moderate and severe neonatal brain hypoxia (HI) models. In their study, inhalation of 2.9% hydrogen did not decrease the infarction volume and brain lipid peroxidation, but there was a trend suggesting a beneficial effect on middle cerebral artery occlusion in adult rats. These discrepancies might be attributed to differing experimental conditions (different degrees of HI insult, age of pups, concentration of hydrogen, and length of hydrogen exposure). Additionally, Sato et al [197] reported that administering hydrogen-rich water to vitamin C-depleted SMP30/GNL knockout mice during both hypoxia and regeneration markedly decreased the levels of hydroxyl and superoxide in brain slices. Li et al [198], examined if hydrogen-rich saline reduced amyloid β (Aβ)-induced neural inflammation, as well as learning and memory deficits in a rat model. The results showed that intraperitoneal injections of hydrogen-rich saline, which were administerd daily once for 2 consecutive weeks, could improve the cognitive and memory functions in rat models with amyloid β 1-42-induced Alzheimer's by preventing neuro-inflammation and oxidative stress. In a middle cerebral artery occlusion model, administering injections of 50% dextrose, and hydrogen gas during the reperfusion (for 2 h) reduced brain infarction, hemorrhagic transformation, and improved neurological function in rats [199].

**Table 3:** Summary of Neuroprotective Effect of Hydrogen

Model	Intervention	Results	Reference
MCAO	1%, 2%, 4% hydrogen during the occlusion (85 min), or reperfusion (35 min) or occlusion + reperfusion (120 min)	Inhalation of hydrogen markedly suppresses brain injury by buffering the effects of oxidative stress. 2% hydrogen is more effective than 4% and 1% hydrogen	[190]

neonatal HI	2% hydrogen (30, 60 and 120 min) or hydrogen saturated saline (5 ml/kg immediately and 8 h after insult)	Hydrogen treatment significantly reduces the apoptotic cells, suppresses caspase-3 and -12 activities, reduces MDA and Iba-1 levels, and improves the long-term neurological and neurobehavioral functions	[194,195]

newborn pig asphyxia	2.1% H_2_-supplemented room air for 1 h and additional 3 h	H_2_-RA ventilation significantly increases cerebrovascular reactivity to hypercapnia after asphyxia/reventilation; no affects on ROS-dependent cerebrovascular reactivity to NMDA	[209]

neonatal HI MCAO	Inhalation of 2.9% hydrogen	Inhalation of 2.9% hydrogen did not decrease the infarction volume and brain lipid peroxidation, but there was a trend suggesting a beneficial effect on MCAO in adult rats	[196]

hypoxia-reoxygenation of brain slices of vitamin C-depleted SMP30/GNL knockout mice	hydrogen-rich pure water	Hydrogen-rich pure water acts as an anti-oxidant and prevents superoxide formation	[197]

amyloid-β-induced Alzheimer's disease	Intraperitoneal hydrogen rich saline (5 ml/kg daily for 2 weeks)	Hydrogen-rich saline prevents beta-induced neuroinflammation and oxidative stress, which may contribute to the improvement of memory dysfunction in this rat model	[198]

MCAO	Inhalation of 2.9% hydrogen during reperfusion	Inhalation of hydrogen during 2 h reperfusion was found to reduce brain infarction, hemorrhagic transformation, and improve neurological function	[199]

chronic physical restraint in mice	Oral intake of hydrogen supplemented water up to 8 weeks	Hydrogen water reduces oxidative stress in the brain, and prevents the stress-induced decline in learning and memory caused by chronic physical restraint	[200]

MPTP induced Parkinson's disease model	Oral intake of hydrogen containing water for 28 days	Drinking hydrogen-containing water significantly reduces the loss of dopaminergic neurons accompanied by significant reduction of oxidative stress which was demonstrated by a significant decrease of DNA damage and lipid peroxidation.	[201]

6-OHDA induced Parkinson's disease model	Oral intake of hydrogen containing water before and after surgery	Prevent both the development and progression of the nigrostrital degeneration and dopaminergic cell loss	[202]

Senescence-accelerated mice	Oral intake of hydrogen containing water for 30 days and 18 weeks	Prevented age-related declines in cognitive ability increases brain serotonin levels and elevates serum antioxidant activity at 30 days while inhibiting neurodegeneration in the hippocampus at 18 weeks	[203]

Oral intake of hydrogen-supplemented water is an alternative mode of hydrogen delivery. Nagata et al [200] tested a hypothesis that drinking hydrogen water *ad libitum*, instead of inhaling hydrogen gas, prevents cognitive impairment by reducing oxidative stress. Their findings demonstrated that the consumption of hydrogen water could prevent the stress (chronic physical restraint)-induced decline in learning and memory in a mouse model via reduction of oxidative stress. In a 1-methyl-4-phenyl-1,2,3,6-tetrahydropyridine (MPTP)-induced Parkinson's disease model, Fujita et al showed that drinking hydrogen-containing water (even as low as 0.04 mM) significantly reduced the loss of dopaminergic neuron. Additionally, reductions in oxidative stress were demonstrated by a significant decrease of 8-oxoguanine (8-oxoG), a marker of DNA damage, and 4-hydroxynonenal (4-HNE), a marker of lipid peroxidation [201]. In a rat Parkinson's disease model induced by intrastriatal injection of catecholaminergic neurotoxin 6-hydroxydopamine (6-OHDA), Fu et al found that oral intake of hydrogen water could prevent both the development and progression of nigrostrital degeneration, and pre-and post-treatment with hydrogen prevented dopaminergic cell loss [202]. Therefore, they concluded that hydrogen water was likely to retard the development and progression of Parkinson's disease. Gu et al investigated the efficacies of drinking hydrogen water to prevent spatial memory decline and age-related brain alterations. Treating senescence-accelerated prone mouse 8 (SAMP8) with hydrogen water for 30 days was found to prevent age-related declines in cognitive ability, which were associated with increased brain serotonin levels, elevated serum antioxidant activity, and inhibition of neurodegeneration in the hippocampus [203].

### Argon

The neuroprotective effects of argon have been confirmed in both *in vivo *and *in vitro *studies. One study led by Ryang et al., analyzed the possible neuroprotective role of argon post-treatment in an *in vivo *rat model of acute focal cerebral ischemia [204]. They found that animals breathing spontaneously 50 vol % argon 1 hr after induction of transient middle cerebral artery occlusion for 1 hr by face mask showed significantly reduced infarct volumes and composite adverse outcomes. Similarly, Loetscher et al. showed using *in vitro *studies that argon post-treatment could reduce damages following oxygen-glucose deprivation and traumatic brain injury [205]. Although both studies along with various other studies [206] published in the literature have shown favorable support for Argon's neuroprotective characteristics, evidence is lacking with regards to the exact mechanism and more studies are required to clarify these mechanisms.

## Conclusion

Although the protective effects of the aforementioned gases have been extensively investigated as a therapeutic option following various neurobiological diseases and events, the definite efficacy and mechanisms of action of these gases should be further validated in more well-designed, clinical trials.

## Competing interests

The authors declare that they have no competing interests.

## Authors' contributions

WL-Role included study design, conduct of the study, data collection and analysis. NHK-Role included study design and manuscript preparation. AS-Role included study design and manuscript preparation. JHZ-Role included study design, conduct of the study, data collection and analysis. All authors read and approved the final manuscript.
